# Blunt Abdominal Trauma in the Third Trimester: Eight Departments, Two Patients, One Survivor

**DOI:** 10.7759/cureus.16688

**Published:** 2021-07-28

**Authors:** Bilal Tasneem, Daniel Fox, Shahnaz Akhter

**Affiliations:** 1 Emergency Medicine, Richmond University Medical Center, Staten Island, USA; 2 Research, Richmond University Medical Center, Staten Island, USA

**Keywords:** uterine rupture, fetal demise, emergency medicine and trauma, pregnancy, multi-disciplinary teams, exploratory laparotomy, third trimester

## Abstract

Blunt abdominal trauma is one of the leading causes of non-obstetrics-related deaths during pregnancy, with motor vehicle collision, falls, and assaults being the most common etiologies. While a trauma team plays a central role in the care of a pregnant trauma patient, a multidisciplinary involvement is vital to ensure the safety of the fetus and the mother. This case study will follow the step-by-step multidisciplinary approach utilized for a 37-year-old female in her third trimester who suffered blunt trauma and arrived at a Level 1 trauma center that led to maternal survival but fetal demise. She was initially evaluated by Emergency Medicine and Obstetrics/Gynecology departments for maternal and fetal trauma, by Orthopedics for several fractures including the pubic ramus and sacral ala fractures, as well as by Neurosurgery for a subarachnoid hemorrhage and a subdural hematoma. Subsequently, the following departments were brought on after the patient suddenly became hypotensive with abdominal tenderness to assess for internal bleeding: Interventional Radiology, Trauma, Surgery, and Urology. Retroperitoneal and pelvic hematomas were found to be the source of bleeding during an emergency laparotomy and the decision was made for an emergency caesarian section. The neonatal intensive care unit ultimately could not start the fetal heart. In the days that followed, the neurosurgery department monitored the worsening intercranial bleeds while Psychiatry and Social Work attended to the patient. A proper systematic approach towards a patient in this situation necessitates expertise from multiple fields, and the success of this interplay greatly affects patient outcomes.

## Introduction

Trauma patients are often time sensitive and high-risk cases that require comprehensive protocols to seamlessly apply complicated and/or intensive management. The very cornerstone of these protocols is how to integrate the vast network of departments effectively to provide the best care, and ultimately, bring about the best outcomes. Additional forethought must then be given to the protocols of even more unique cases where there is a larger multidisciplinary need. The literature states that “up to 7% of all pregnancies are complicated by trauma” [1]. A situation that highlights the need for a multidisciplinary approach is an abdominal injury in a pregnant patient, where services including but not limited to Emergency Medicine, Obstetrics/Gynecology (Ob/Gyn), and General Surgery are necessary for proper patient care. The following case study further required the expertise of Orthopedics, Pediatrics, Urology, Interventional Radiology (IR), Social Services, and Psychiatry. With little national data focused on the maternal and fetal survival ratio on third-trimester patients, we report a case of blunt abdominal trauma in a 27-week pregnant 37-year-old female, which resulted in maternal survival but fetal demise in the hopes to create a better understanding of the situation leading to an increase in maternal and fetal survival rates.

Background

Trauma during pregnancy is the leading cause of non-obstetrics-related mortality: “It is also the number one cause of fetal demise” [2]. Trauma in pregnancy is categorized into three different types by the American College of Obstetricians and Gynecologists (ACOG): blunt abdominal trauma, pelvic fracture, and penetrating trauma [3].

According to the “Guidelines for the Management of a Pregnant Trauma Patient”, printed by the Society of Obstetricians and Gynecologists of Canada, the obstetrician “plays a major role in determining gestational age, optimizing uteroplacental perfusion, assessing fetal wellbeing, providing information about the risk of radiation exposure and use of medications, and deciding upon and executing an emergency Caesarean section” [4]. A pregnant trauma patient should receive special attention pertaining to fetal evaluation when gestational ages is greater than or equal to 23 weeks, and to possible pregnancy-specific trauma complications like placental abruption [4]. Traumatic uterine rupture is another possible complication in the setting of high-energy abdominal trauma consistent with motor vehicle accidents. Both placental abruption and traumatic uterine rupture require prompt diagnosis given their quick onset, progression, and life-threatening complications [5]. Implementing the emergency triage protocol in the management of pregnant trauma patients can facilitate a smoother multidisciplinary action to ensure the highest quality of patient care.

## Case presentation

A 37-year-old female in her 27th week of pregnancy was hit by a motor vehicle and brought to a Level 1 trauma center at 13:30. Trauma protocol was activated in house after Emergency Medical Services notification upon arrival to the Emergency Department (ED). On first evaluation, the patient was confused, agitated, and highly combative. During her transfer to the hospital stretcher, she became violent with the nursing staff and was placed in physical restraints. On initial physical assessment at the ED, the patient was repeating herself with confused speech irrespective of the question asked. Initial vitals were blood pressure 107/73 mm Hg, heart rate (HR) 95 beats per minute, respiratory rate 16, temperature 98.3°F. The nursing staff obtained IV access and completed diagnostic lab work. The Ob/Gyn team was paged immediately.

Primary evaluation showed an intact airway with bilateral (B/L) breath sounds, and B/L brachial and femoral pulse. The patient had a Glasgow Coma Scale (GCS) score of 14, was alert/oriented to person, moved all extremities, with slightly altered mental status while repeating the same words in Spanish. Detailed examination revealed that the patient had bruises on the left forehead above the eyebrow, no chest wall tenderness or spinal tenderness, and extremities were warm and well perfused with no gross deformities. The abdominal sonogram revealed intrauterine pregnancy with a fetal heart rate (FHR) of 140 beats per minute, the placenta was noted to be anterior, and there were no signs of placental rupture. The patient showed no signs of contractions or vaginal bleeding and at that time, the fetus was felt to be stable.

**Figure 1:**
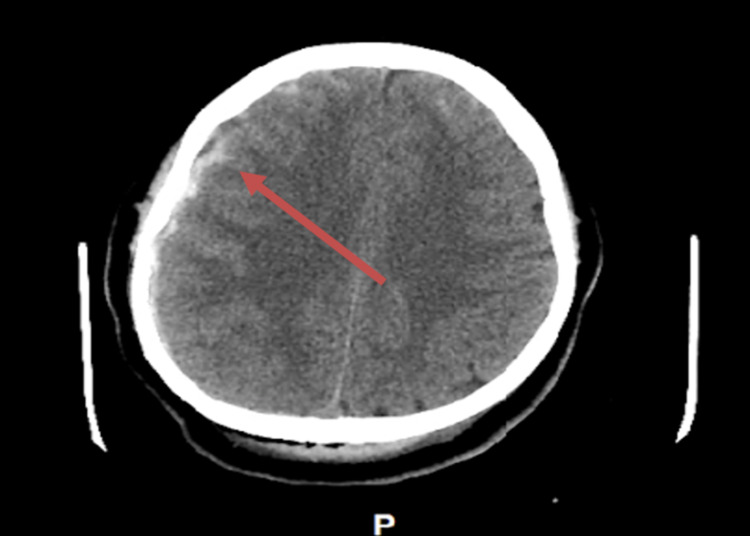
Initial brain CT showing subdural hematoma (arrow)

**Figure 2:**
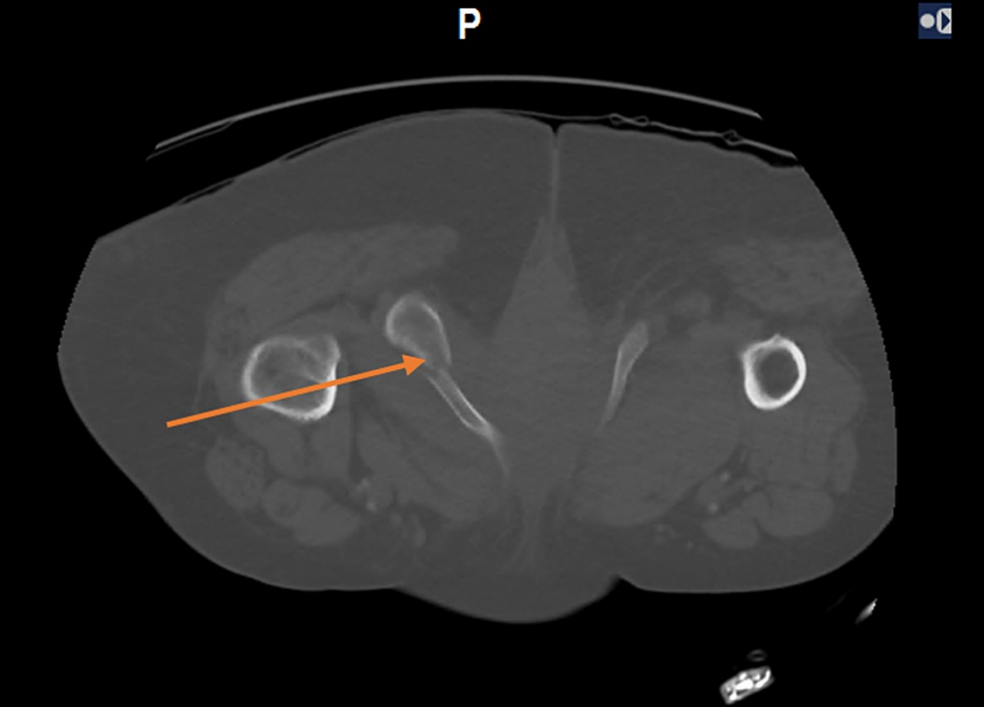
Initial pelvic CT showing an inferior pubic ramus fracture (arrow) of the pelvis

**Figure 3:**
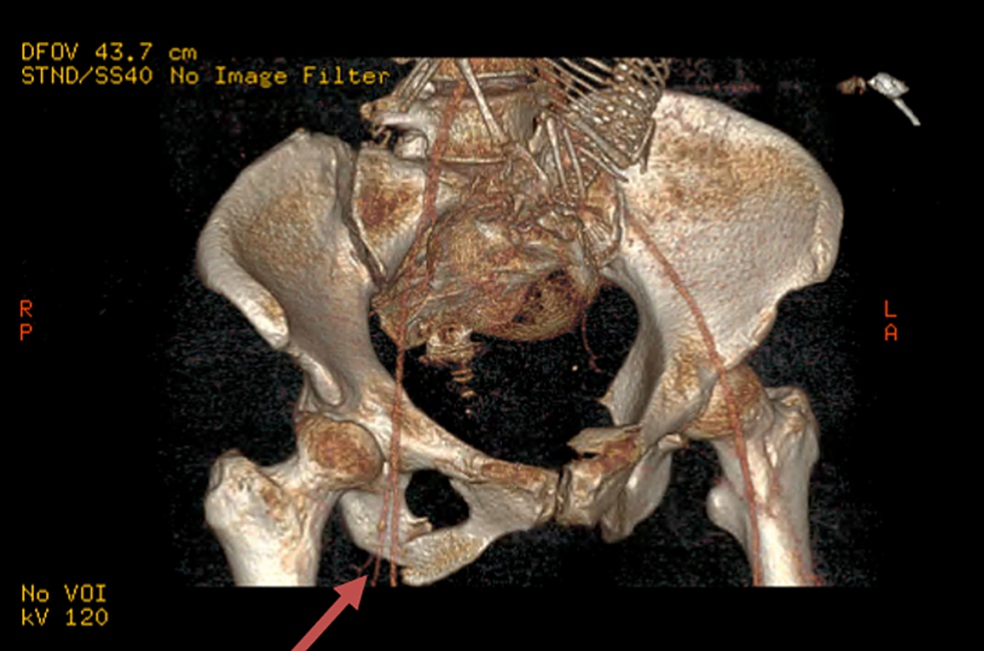
Pelvic CT - 3D reconstruction of the pelvic fracture (arrow)

**Figure 4:**
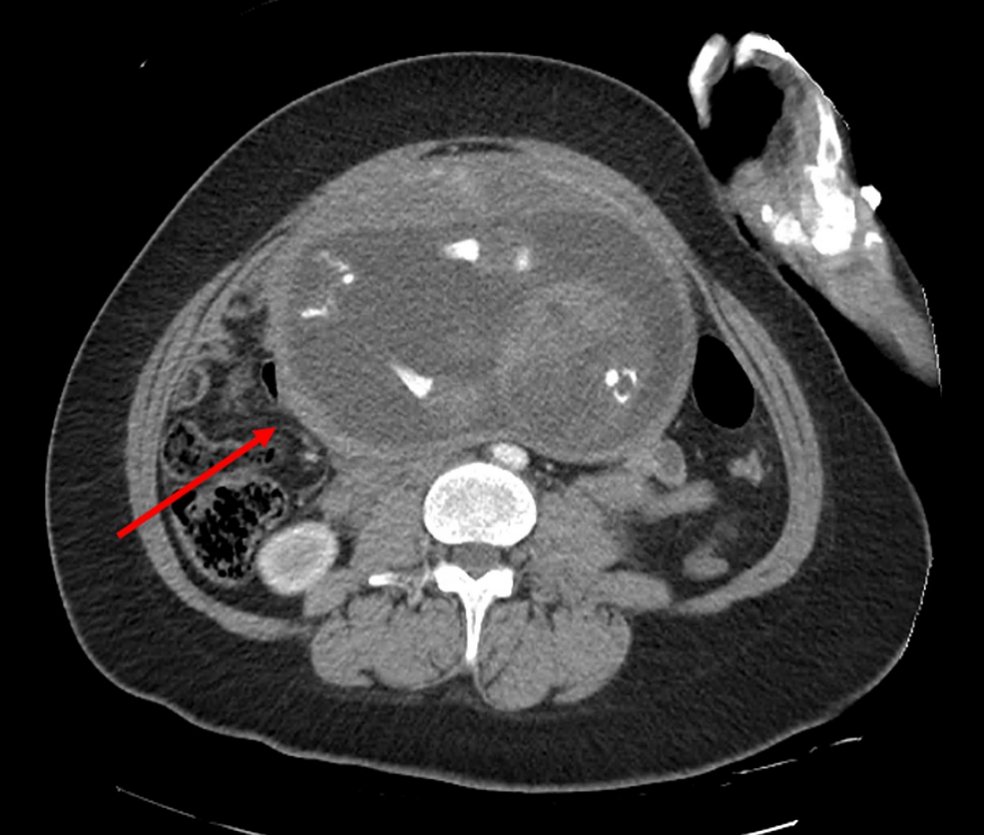
CT scan showing a small abdominal hematoma (arrow)

**Figure 5:**
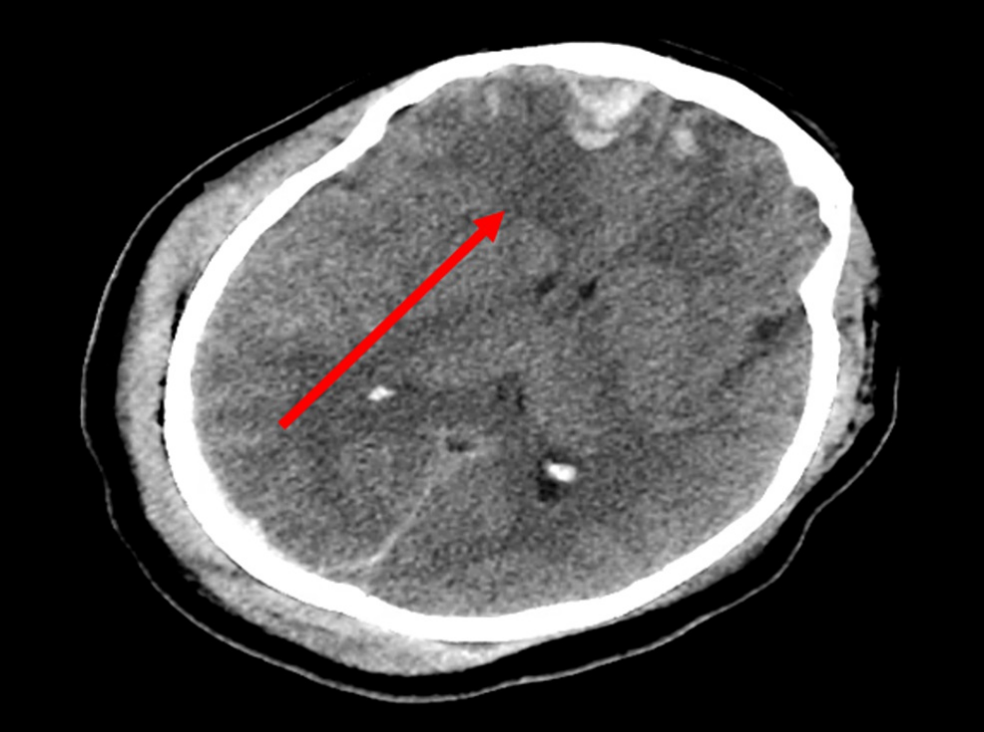
Brain CT six hours after initial presentation showing the progression of intracranial hemorrhages (arrow)

Imaging was then performed including X-ray of the chest and pelvis as well as CT scans of the head, chest, abdomen, and pelvis. Results from the diagnostic blood work were reviewed (Table 1). Preliminary results of the imaging revealed a subarachnoid hemorrhage with a small subdural hematoma, a moderate-sized extraperitoneal ventral pelvic hematoma, a small right retroperitoneal hematoma encasing the intrahepatic inferior vena cava (IVC), bilateral fractures of the pubic ramus and sacral ala, a widening of the pubic symphysis and right sacroiliac joint, and a mildly displaced fracture of the lumber 5 (L5) transverse process (Figures 1-5). The CT images led to consultations with Orthopedics and Neurosurgery departments. Neurosurgery recommended non-operative management with repeat head CT at 19:00, while Orthopedics recommended a pelvic binder and no operative interventions. IR was also consulted but determined there was no active bleeding and no need for embolization.

**Table 1:** Initial CBC, CMP and coagulation panels drawn from the trauma bay on patient arrival CBC, complete blood count; CMP, comprehensive metabolic panel; WBC, white blood cell; RBC, red blood cell; Hgb, hemoglobin; Hct, hematocrit, MCV, mean cell volume; MCH, mean corpuscular hemoglobin; MCHC, mean corpuscular hemoglobin concentration; RDW, red cell distribution width; Plt, platelet; MPV, mean platelet volume; Gran, granulocyte; Neut, neutrophil; Lymph, lymphocyte; Mono, monocyte; Eos, eosinophil; Baso, basophil; Abs immat gran, absolute immature granulocyte; BUN, blood urea nitrogen; GFR, glomerular filtration rate; MDRD, Modification of Diet in Renal Disease; AST, aspartate aminotransferase; ALT, alanine transaminase; PT, prothrombin time; aPTT, activated partial thromboplastin time; INR, international normalized ratio

Blood work	
WBC	20.1
RBC	4.03
Hgb	11.8
Hct	36.1
MCV	89.6
MCH	29.3
MCHC	32.7
RDW	13.2
Plt count	201
MPV	12.1
Immature gran % (auto)	4.8
Neut % (auto)	75.8
Lymph % (auto)	14.9
Mono % (auto)	3.7
Eos % (auto)	0.5
Baso % (auto)	0.3
Abs immat gran (auto)	0.97
Abs Neut (auto)	15.2
Abs Lymph (auto)	3
Abs Mono (auto)	8
Abs Eos (auto)	0.1
Abs Baso (auto)	0.1
Sodium	140
Potassium	3.3
Chloride	109
Carbon dioxide	20
Anion gap	15.3
BUN	9
Creatine	0.6
Estimated GFR (MDRD)	>90.00
Glucose	111
Calcium	9
Total bilirubin	0.5
AST	40
ALT	26
AST/ALT ratio	1.54
Alkaline phosphatase	138
Total protein	7.1
Albumin	2.9
Ethyl alcohol	<3.0
PT	13.9
aPTT	26.8
INR	1.12

By 15:00, the patient had been transferred to the Surgical Intensive Care Unit (SICU) for continued care and observation by both the Trauma team and Obstetrics team. Repeat sonogram was performed by the Ob/Gyn team and showed good FHR and movement. Ob/Gyn attempted to place continued fetal monitors on the patient; however, due to her noncompliance, she never had continuous monitoring placed. The team was able to examine the patient and at that time, described the patient’s abdomen as soft, gravid, nontender/nondistended in all four quadrants with no signs of uterine injury.

Within two hours of placement in the SICU, the patient became tachycardic and hypotensive with the HR 150/minute and systolic blood pressure 70 mm Hg with abdominal tenderness. The patient was immediately transferred to the operating room (OR) and had an emergency laparoscopy for the evaluation of internal bleeding as well as an emergency C-section.

During Foley placement, the patient was found to have gross hematuria and Urology consult was obtained. It was found that the gross hematuria was due to an extraperitoneal bladder injury from the pelvic fracture. A chromocystogram performed in the OR found no extravasation of fluids into the peritoneal cavity seen, whereby Urology ruled out an intraperitoneal injury of the bladder. Upon review of the CT scan, the Surgery team noted no hydronephrosis or intraparenchymal damage, nor appreciated any functional renal damage. The Surgery team proceeded with the exploratory laparotomy and after initial incision, approximately 200 ml of blood was observed throughout the abdominal pelvic cavity, and three pelvic hematomas were located on the pelvic side wall and bladder. There was no active bleeding noted and it was determined that the bleeding source was the retroperitoneal hematoma. The Ob/Gyn and trauma team also noted multiple contusions, abrasions, and superficial lacerations of the uterus along the posterior cul de sac. They also noted a 2-cm uterine rupture about 4-5 cm below the fundus midline. The OB team performed an emergency C-section and the patient received RBC transfusion that stabilized her vitals. During further examination, the Ob team found superficial lacerations throughout the uterine surface but no active bleeds from any uterine vasculature, and the uterine fundus was firm. The placenta was protruding through the incision site but was removed intact.

The neonate was delivered via C-section, and the baby had pale limbs, no heart rate, and was not moving. The neonatal intensive care unit (NICU) team started resuscitation maneuvers. Despite full resuscitative efforts, the neonatal heart remained in asystole and the time of death was reported as 18:11.

**Figure 6:**
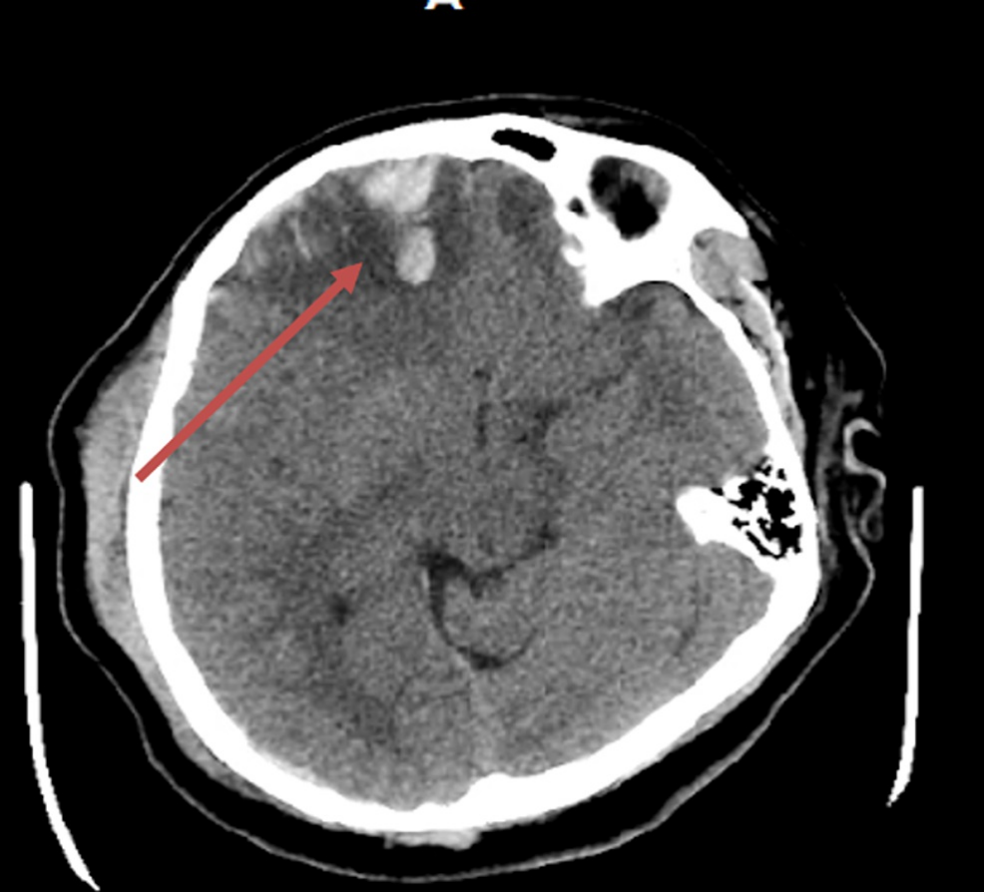
Brain CT at 48 hours showing the worsening progression of intracranial injuries (arrow)

Post C-section and exploratory laparotomy, the patient was closed and placed back in the SICU under mechanical ventilation, where she became hemodynamically stable. Intra-operative lab work was performed and showed hemoglobin at 7 and platelet count 80. The patient was given six units of packed red blood cells, four units of fresh frozen plasma, and two units of platelets. The patient received a Camino intracranial pressure monitor adapter that was placed at bedside by the Neurosurgery team in the SICU. After stabilization, the patient eventually underwent surgical repair of her pelvic fractures and was monitored closely by the Neurology and Trauma teams for several more days. Repeat head CTs showed worsening intracranial injuries (Figures 5, 6). During her hospital stay, the patient was seen by Social Work and Psychiatry teams and both the patient and family received counseling and resources for post-traumatic stress and to help grieve for the child. The mother's mental and physical status showed positive progression during her follow-up appointments at the hospital.

## Discussion

In 2001 to 2005, the National Trauma Data Bank published a report that found “trauma related mortality among pregnant women [to be lower] than that among non-pregnant women” [4]. This difference has been attributed to the fact that pregnant women are less likely to engage in dangerous activities: “the protective hormonal, and physiological effects of pregnancy as well as a higher likelihood of hospital admission of pregnant versus non pregnant trauma victims” [4].

Trauma in pregnancy presents a series of unique concerns that requires a multi-disciplinary approach to ensure appropriate care. Specifically in the case of a rare uterine rupture, when there is a direct insult to abdomen, “the risk for maternal and/or fetal injury is 10%-15% in first trimester, up to 40% in second trimester, and up to 54% during the third trimester” [2]. In this case, the mother in her third trimester required care from Obstetrics/Gynecology, Emergency Medicine, Orthopedics, Trauma Surgery, Neurosurgery, NICU, Urology, Interventional Radiology, Psychiatry, and Social Services. It is vital that these patients be identified early and if possible, brought to a tertiary medical center where optimal integrated care can be provided.

In this particular case study, we see two out of the three subdivisions for trauma in pregnancy that are defined by the ACOG. Since providers are faced with dealing with two individuals, mother and fetus, the management of pregnant patients who suffer from trauma should include members of multiple disciplines to optimize care. While saving both individuals is always the goal, it is crucial to remember that the priority is maternal stability and survival. Hence, like nonpregnant patients, “initial resuscitation and management of a pregnant trauma patient should be consistent with the advanced trauma life support protocols, which follow the ABCDE pattern: airway, breathing, circulation, and disability, and exposure” [3]. On top of that, if a long blackboard is utilized for patient transport, the board must be tilted at a 15° angle with elevation of the right hip [2]. The tilt avoids the complication of vena cava compression by the enlarged uterus that can cause hypotension leading to up to “30% decrease in cardiac output” that can have lethal consequences [2]. Thus, any pregnant trauma patient needs to start off with the Surgery, Emergency Medicine, Ob/Gyn, Nursing, and Technician teams at bedside on arrival with other services on hand to join the team should any further injury or necessity arise.

As previously discussed, the clinical assessment for a pregnant patient is complicated by anatomical and physiological changes to the pregnant mother as well as the care of the fetus, making a trauma situation even more complex. During pregnancy, there is an increase in progesterone that relaxes smooth muscle and lowers “blood pressure by up to 15 mmHg” [2]. Additionally, there is a rise in estrogen levels that can result in heart rates increased by “up to 15 beats per minute”, as well as an increase in “cardiac output by 30% to 50%” [6]. Another unique physiologic change during pregnancy is that “maternal blood volume increases by 30% to 50%” [2]. The sum of all these changes leads to a “hyperdynamic hypervolemic state [which] makes the assessment for shock challenging because the mother may not show signs of distress until she hemorrhages 1500 to 2000ml” [2]. After 2500 ml of blood loss, the mother’s condition can rapidly deteriorate [6]. Once shock develops, the chance of saving the fetus is 20%, thus making it vital to involve Ob/Gyn early to immediately assess age/viability and status of the pregnancy/fetus [2]. An emergency C-section performed after 25 weeks of gestation for specific indications following trauma is associated with 45% fetal survival and 72% maternal survival; if the caesarian section and delivery are done within five minutes, there is an excellent probability of survival, but it is more unlikely as time goes on [7]. Underpinning all of this clinical management is prioritizing the mother’s health; however, ensuring that all subspecialties required are consulted early will increase the chances of both the mother and fetus surviving.

As seen in this case study, despite optimizing care and involving all services necessary, patients may still experience negative outcomes and fetal demise. In this case, even with the fetus being stable on arrival, it is felt that the fetus expired due to maternal shock and instability. Even though a 2-cm uterine tear was eventually found during the emergency caesarian section, the question becomes, “Would an earlier obstetrics sonogram in the trauma bay have noted a decrease in the amniotic fluid index?,” hinting at a tear in the uterus and thereby affected management. Providers are often faced with this difficult decision of choosing to conduct further testing in the ED, taking the patient to the OR for emergent surgical exploration, or transferring the patient to the ICU for observation, Both the “wait and see” and “take immediate action” trains of thought hold their own level of risk to the patient; however, physicians are trained to give patients the best chance of survival, which varies from case to case. Navigating these waters of clinical judgment to lead to the best course of action for the patient requires integration of all the specialties involved. Effectively consolidating and acting upon the specific expertise they bring allows for the best elucidation of the patient’s overall picture and ultimately, prognosis.

It is also important to note that care for the mother does not stop with physical injuries. The sensitivity of post-traumatic stress and the long-term effects of losing a child should be approached with care and is best managed by specialists in social services and psychiatry.

A multidisciplinary post-event meeting was held after the case to discuss the details in full. It was determined that all teams and specialties involved showed a quick response time and an excellent eye for detail during this case.

## Conclusions

Each situation in the trauma bay can be inherently different based on that patient's current physiology and past medical history. By instating proper protocols, we give medical professionals a baseline at which to begin so that they can focus on each individual patient, giving each a better chance at survival. While percent chance of survival for both parties can be less than 50% in an abdominal trauma in the third trimester, with proper multidisciplinary protocols, training, and coordination, we can give our patients the highest chance at survival.

## References

[1] Petrone P, Talving P, Browder T, Teixeira PG, Fisher O, Lozornio A, Chan LS (2011). Abdominal injuries in pregnancy: a 155-month study at two level 1 trauma centers. Injury.

[2] Mowry M (2017). Case study: obstetrical trauma with maternal death and fetal survival. Crit Care Nurs Q.

[3] Mirza FG, Devine PC, Gaddipati S (2010). Trauma in pregnancy: a systematic approach. Am J Perinatol.

[4] Jain V, Chari R, Maslovitz S (2015). Guidelines for the management of a pregnant trauma patient. J Obstet Gynaecol Can.

[5] Suchecki G, Tilden H, Roloff K, Chandwani D, Neeki M (2020). Management of traumatic uterine rupture in blunt abdominal trauma: a case report and literature review. Cureus.

[6] Muench MV, Canterino JC (2007). Trauma in pregnancy. Obstet Gynecol Clin North Am.

[7] Morris JA Jr, Rosenbower TJ, Jurkovich GJ (1996). Infant survival after cesarean section for trauma. Ann Surg.

